# Deep learning based screening model for hip diseases on plain radiographs

**DOI:** 10.1371/journal.pone.0318022

**Published:** 2025-02-13

**Authors:** Jung-Wee Park, Seung Min Ryu, Hong-Seok Kim, Young-Kyun Lee, Jeong Joon Yoo

**Affiliations:** 1 Department of Orthopaedic Surgery, Seoul National University College of Medicine, Seoul, South Korea; 2 Department of Orthopaedic Surgery, Seoul National University Bundang Hospital, Seongnam, South Korea; 3 Department of Orthopaedic Surgery, Seoul Medical Center, Seoul, Republic of Korea; 4 Department of Orthopaedic Surgery, Seoul National University Hospital, Seoul, South Korea; Humanitas Clinical and Research Center - IRRCS, ITALY

## Abstract

**Introduction:**

The interpretation of plain hip radiographs can vary widely among physicians. This study aimed to develop and validate a deep learning-based screening model for distinguishing normal hips from severe hip diseases on plain radiographs.

**Methods:**

Electronic medical records and plain radiograph from 2004 to 2012 were used to construct two patient groups: the hip disease group (those who underwent total hip arthroplasty) and normal group. A total of 1,726 radiographs (500 normal hip radiographs and 1,226 radiographs with hip diseases, respectively) were included and were allocated for training (320 and 783), validation (80 and 196), and test (100 and 247) groups. Four different models were designed–raw image for both training and test set, preprocessed image for training but raw image for the test set, preprocessed images for both sets, and change of backbone algorithm from DenseNet to EfficientNet. The deep learning models were compared in terms of accuracy, sensitivity, specificity, positive predictive value (PPV), negative predictive value (NPV), F1-score, and area under the receiver operating characteristic curve (AUROC).

**Results:**

The mean age of the patients was 54.0 ± 14.8 years in the hip disease group and 49.8 ± 14.9 years in the normal group. The final model showed highest performance in both the internal test set (accuracy 0.96, sensitivity 0.96, specificity 0.97, PPV 0.99, NPV 0.99, F1-score 0.97, and AUROC 0.99) and the external validation set (accuracy 0.94, sensitivity 0.93, specificity 0.96, PPV 0.95, NPV 0.93, F1-score 0.94, and AUROC 0.98). In the gradcam image, while the first model depended on unrelated marks of radiograph, the second and third model mainly focused on the femur shaft and sciatic notch, respectively.

**Conclusion:**

The deep learning-based model showed high accuracy and reliability in screening hip diseases on plain radiographs, potentially aiding physicians in more accurately diagnosing hip conditions.

## Introduction

Hip joint-related pain is widely recognized, affecting both young athletes and elderly patients, and can cause severe functional disabilities [1–3]. Among the various causes of hip pain, diseases such as osteonecrosis of the femoral head (ONFH) and osteoarthritis of the hip can lead to loss of ambulatory function if not treated appropriately [4,5]. A bedridden state due to these conditions can result in numerous complications, including aspiration pneumonia, pressure sores, venous thromboembolism, pulmonary embolism, and major adverse cardiac events (MACE) [6–10].

However, diagnosing the cause of hip pain is often very challenging. Because the hip joint is located deep beneath thick layers of large muscles, the pain is frequently referred to the groin, trochanter, or buttock regions [11]. In addition, the differential diagnosis of hip pain widely varies among different conditions, and even orthopedic surgeons often struggle to diagnose accurately [12–15]. In a retrospective study of 150 patients, over 80% were misdiagnosed by primary physicians [16]. Interestingly, the study also found that the problems of lumbar spine such as spinal stenosis were the third most common cause of hip pain [16]. Conversely, Nakamura et al. found that 8% of patients with ONFH and 17% of those with osteoarthritis of hip exhibited low back pain [17,18]. In clinical settings, many patients who have problems in the hip joint are commonly misdiagnosed as simple muscle sprain, spine problems, or arthrosis of other joints even with the plain hip radiographs taken. These findings underline the importance of screening model in patients with hip pain to distinguish whether the hip joint should be thoroughly investigated.

For the diagnosis of hip diseases, plain hip radiographs remain to be the keystone. The plain radiographs are usually the first imaging test to be performed in patients with hip pain. However, the accuracy of plain hip radiographs in diagnosing the specific hip diseases is known to be limited, demanding the use of more advanced imaging techniques [19]. Although contemporary imaging modalities such as MRI or CT scans are more sensitive, they are not currently routinely recommended because they are expensive and time-consuming. Moreover, the high acquisition and maintenance costs of these advanced modalities limits their use in developing countries or rural areas [20–22].

With the advent of artificial intelligence (AI) technologies, the potential practicality of plain hip radiographs could be revisited. Deep learning algorithm, notably convolutional neural network (CNN) model, has displayed high performance in medical image classification task through supervised learning [23,24]. Although plain hip radiographs are difficult to provide final and specific diagnosis, the high availability of plain radiographs makes them a practical choice for screening hip disease with the aid of the CNN.

Previous studies have focused on detecting specific diseases or conditions using deep learning models on radiographs. However, to the best of our knowledge, no study has developed a screening model for a wide range of hip diseases using plain radiographs and deep learning algorithms. Therefore, the purpose of this study was to develop and validate a deep learning-based screening model to distinguish normal hips from those with significant pathological changes, using plain radiographs as a preliminary step toward diagnosing severe hip diseases. This study aims to contribute to the field by developing a novel deep learning-based screening model for detecting hip diseases using plain radiographs, providing a practical tool that can be applied in clinical settings. Unlike previous studies that focused on specific conditions, this model is designed to screen a wide range of hip diseases with high accuracy.

## Materials and methods

The study is organized into several key phases: data collection, model development, model training using different algorithms, and validation using external data. Each phase is designed to ensure that the model is robust and generalizable across different clinical settings.

### Datasets

This study was conducted as a multicenter study on two tertiary hospitals. The institutional review board of the local medical institution approved the design and protocol of this retrospective study (No B-2307-843-106) and waived the requirement for informed consent. The radiographs were retrospectively collected in both centers. The deep learning model was constructed using the data from one hospital and the external validation was performed in the other hospital. The normal hip radiographs were identified using a clinical data warehouse (CDW) database. The electronic medical record (EMR) program was adopted in 2004 in both hospitals and CDW database covers all the radiographic data from 2004. Eight hundred ninety-seven patients who had plain hip anteroposterior (AP) radiographs taken between 2004 and 2012, with radiologists’ reports indicating "no significant bony abnormality," "no bony abnormality," "no specific abnormal findings," "within normal limits," or "no definite bony lesion," were identified. Two of the authors (JWP, SMR) reviewed all radiographs and only included normal radiographs, excluding the patients who received previous hip surgery (n = 174). Among 723 radiographs, we selected 500 radiographs for analysis in the control group.

One thousand six hundred and thirty one patients who had hip diseases were identified as those who received total hip arthroplasty (THA) from 2004 to 2012 in the same institution. Hip radiographs of these patients were reviewed by two of the authors (JWP, SMR). Patients who received bilateral THA (n = 257) and who had prior hip surgery (n = 148) were excluded. A total of 1,226 radiographs with hip disease were included for the screening model construction.

The dataset was stratified as follows: training set (320 normal, 783 hip disease), validation set (80 normal, 196 hip disease), and test set (100 normal, 247 hip disease). To address the class imbalance issue, we employed PyTorch’s ImbalancedDatasetSampler during the training and validation phases. This approach facilitated oversampling of the minority class and undersampling of the majority class, ensuring balanced representation of both normal and abnormal cases in each epoch.

### Model training

For model training, two authors (JWP, SMR) reviewed all radiographs and confirmed the diagnosis whether the radiograph showed evidences of hip diseases or was normal. Furthermore, they confirmed the diagnosis of each patient with their respective diagnosis on the radiographic findings and data from the EMR. The diagnoses of the included patients were listed in Table 1. The binary classification model (hip disease vs normal) was constructed through supervised learning. Among the total of 1,726 hip radiographs, training set, validation set, and test set included 1,103, 276, and 347 radiographs, respectively. Training set was used for the training of the screening model, while validation set was used for tuning to enhance the model performance, and test set was used for internal validation. The radiograph images were preprocessed by resizing to a standard resolution of 1024x1024 pixels, normalizing the pixel values to a range of 0 to 1.

**Table 1:** Diagnoses of the patients with hip disease.

Diagnosis	N	%
Osteonecrosis of the femoral head	476	40
Dysplastic hip	151	13
Primary osteoarthritis	143	12
Septic hip sequelae	103	9
Legg-Calves-Perthes disease sequelae	89	7
Hip fracture	76	6
Posttraumatic osteoarthritis	42	4
Inflammatory arthritis	40	3
Tumor	32	3
Cerebral palsy sequelae	26	2
Tuberculous arthropathy	22	2
Others	26	2
Total	1,226	100

The neural network architecture employed in this study is based on the EfficientNet-B3 model, pre-trained on the ImageNet dataset. EfficientNet is a family of convolutional neural network models designed for efficient performance and scalability, making it a suitable choice for our medical image analysis task [25]. Notably, the model implementation was conducted using open-source software, specifically Python version 3.8.3, facilitated by libraries such as PyTorch (version 1.7) and MONAI (version 0.6.0), a dedicated library for medical image analysis.

During the training phase, a meticulously crafted algorithm iterated through batches of input data, calculating the loss using binary cross-entropy as the primary metric. This loss was then utilized to update the model parameters through the process of backpropagation, with the Adam optimizer employed for efficient convergence. Data augmentation and early stopping were employed to enhance generalization and prevent overfitting. These computational resources operated within a Linux environment running Ubuntu version 18.04.5 and utilized the CUDA platform version 11.0 from Nvidia for GPU acceleration. The system comprised of a 16-thread Intel Core i9-9900K CPU clocked at 3.60 GHz, complemented by 32 GB of DDR4 Synchronous Dynamic Random Access Memory (DRAM), and powered by an Nvidia GeForce RTX 2080 Ti graphics processing unit (GPU).

### Model development

The screening model was developed through four consecutive versions. In the first model, raw image files (DICOM files) were used for both the training and test sets, and the DenseNet CNN model served as the backbone algorithm for classification. In the second model, images were preprocessed with center-cropping, resizing, and zero padding for training, but raw images were used for testing. The third model utilized preprocessed images for both the training and test sets. In the fourth model, the backbone algorithm was changed from DenseNet to EfficientNet, and a nonlocal block network was added to the algorithm [25–27]. The specific codes to train and develop the models are listed in the following link.

(https://github.com/smryuphd/Hip2D_SNUBH/blob/main/Binary_Classifier_240807_EN_NLB_github.ipynb)

Our final model utilizes an EfficientNet-B3 backbone followed by a nonlocal block. The input to the model is a preprocessed image of size 512x512x3. The EfficientNet-B3 backbone consists of multiple mobile inverted bottleneck convolutional (MBConv) blocks, which efficiently balance model size and accuracy. This backbone extracts hierarchical features from the input image. Following the EfficientNet-B3 backbone, we implement a nonlocal block. This block is designed to capture long-range dependencies in the feature space, which is particularly beneficial for analyzing complex medical images like hip radiographs. The output from the nonlocal block is then passed through a global average pooling layer to reduce spatial dimensions, followed by a fully connected layer with sigmoid activation for binary classification (S1 Fig., S1 File).

### External validation

The external validation was performed using the data from the other hospital. The radiographs of the normal group and the hip disease group were collected in the same manner as the data obtained to construct the model. Both normal and disease groups were selected from the hip radiographs of the patients who were treated between 2004 and 2012 for hip pain. The hip disease group consisted of patients who received THA and were selected ensuring a representative sample of the disease population. This approach allowed us to capture the full spectrum of disease severity and progression, while minimizing potential selection biases. Two hundred radiographs were selected from each group, respectively. The final (4^th^) screening model was externally validated in this cohort.

### Statistical analysis

The best-performing model was selected based on the validation loss on the validation dataset, with the optimal epoch determined by the lowest validation loss. The area under the receiver operating characteristic curve (AUROC) was calculated using the predicted values and true labels. The Youden index was then used to determine the optimal threshold, which facilitated the calculation of accuracy, sensitivity, specificity, positive predictive value (PPV), negative predictive value (NPV), and F1-score [28].

$$Accuracy=\frac{TP+TN}{TP+TN+FP+FN}$$

$$Sensitivity\;(recall)=\frac{TP}{TP+FN}$$

$$Specificity=\frac{TN}{TN+FP}$$

$$PPV=\frac{TP}{TP+FP}$$

$$NPV=\frac{TN}{TN+FN}$$

$$F1-score=2\times \frac{Precision\times Recall}{Precision+Recall}$$

AUROC was calculated using the trapezoidal rule

Where TP = true positives, TN = true negatives, FP = false positives, FN = false negatives, PPV = positive predictive value, NPV = negative predictive value, AUROC = area under the receiver operating characteristic curve.

To improve the evaluation process, Gradient-weighted Class Activation Mapping (Grad-CAM) was used to highlight the areas of the input that had the most impact on the model’s decision-making [29].

## Results

### Patient demographics

Plain hip radiographs of 1,226 patients in hip disease group and 500 patients in normal group were included for the development of screening model (Table 2).

**Table 2:** Patient demographics.

Demographics	Hip disease (n = 1,226)	Normal (n = 500)
Age (mean ± SD), years	54.0 ± 14.8	49.8 ± 14.9
Sex, N (%)		
Men	603 (49%)	185 (37%)
Women	623 (51%)	315 (63%)

### Performance of each screening model

**Fig 1:**
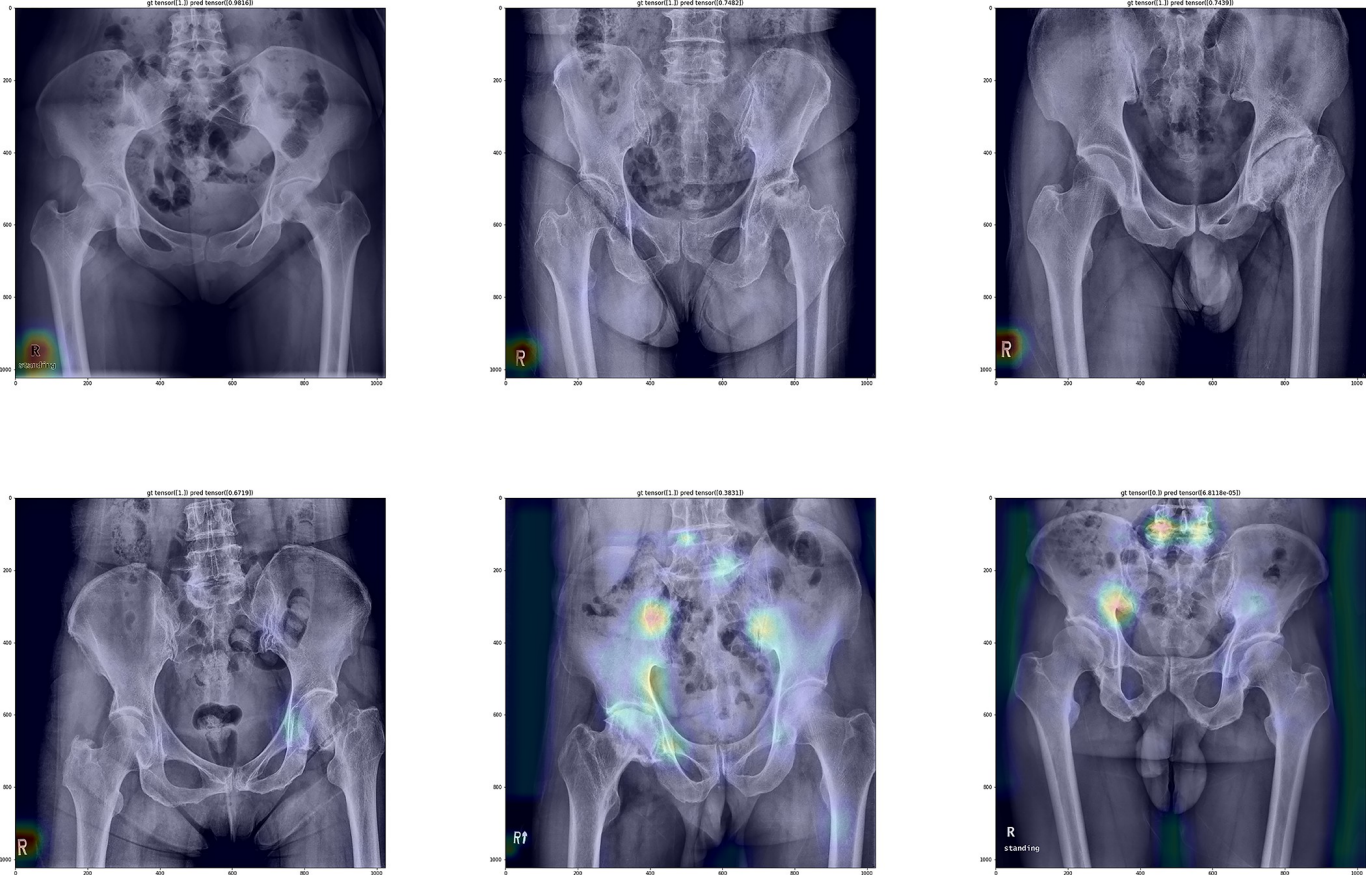
The Grad-CAM images of the 1^st^ model showing the emphasis on the unrelated marks (“stand” or “R”).

The result of first model showed accuracy, sensitivity, specificity, and F1-score of 0.96, 0.96, 0.98, and 0.97, respectively. However, the Grad-CAM image showed that the screening model trained using the unrelated marks such as “stand” or “R” (indicating right side) from the uncropped radiograph (Fig 1).

**Fig 2:**
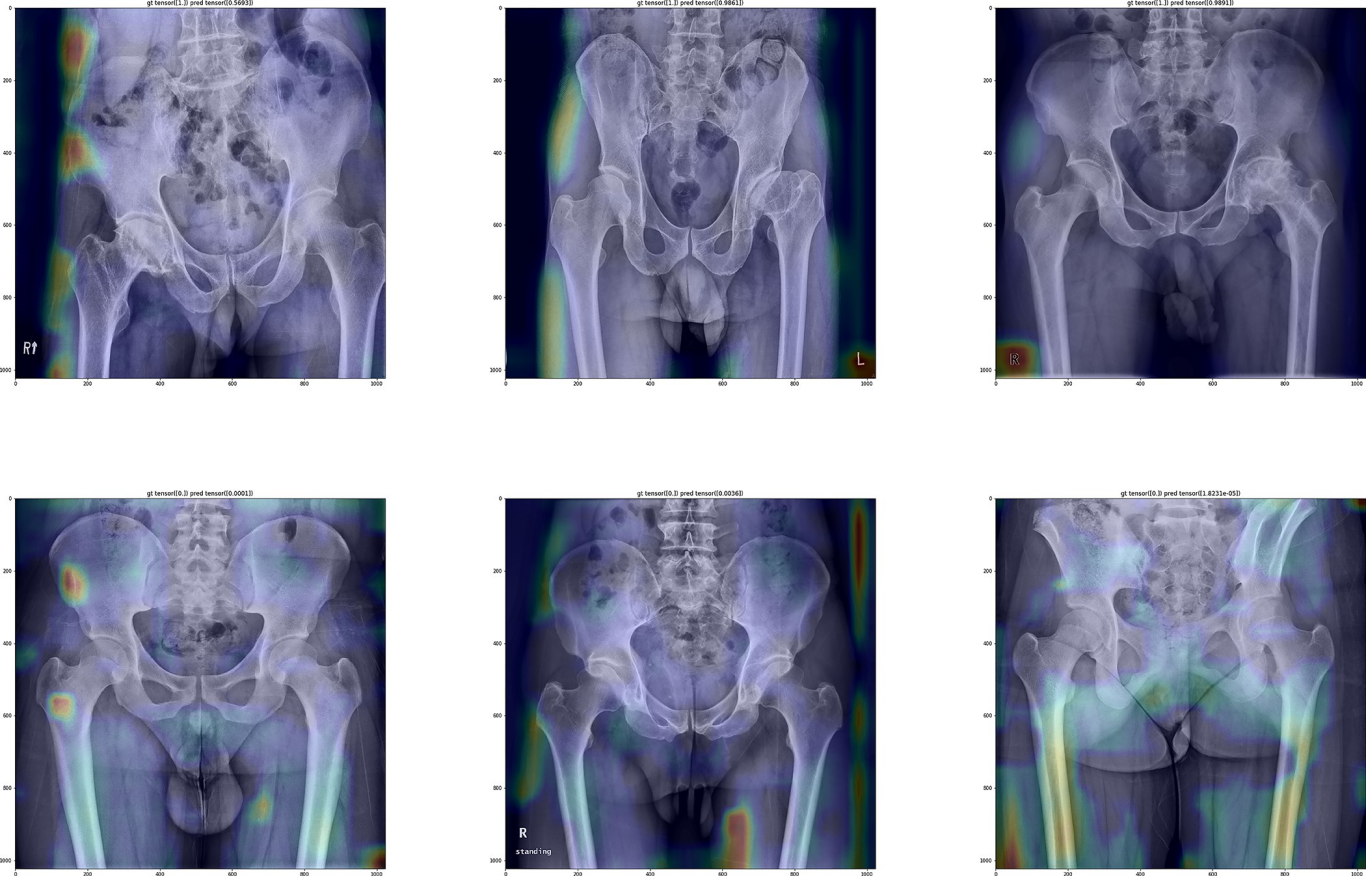
The Grad-CAM images of the 2^nd^ model showing the emphasis on the femoral shafts.

The second model in which the preprocessed radiographs were used for the training set but raw radiographs were used for the test set showed the accuracy, sensitivity, specificity, and F1-score of 0.93, 0.90, 0.99, and 0.94, respectively. The performance decreased compared to the first model and the gradcam image showed that the second model mainly focused on the femur shaft rather than the hip joint (Fig 2).

**Fig 3:**
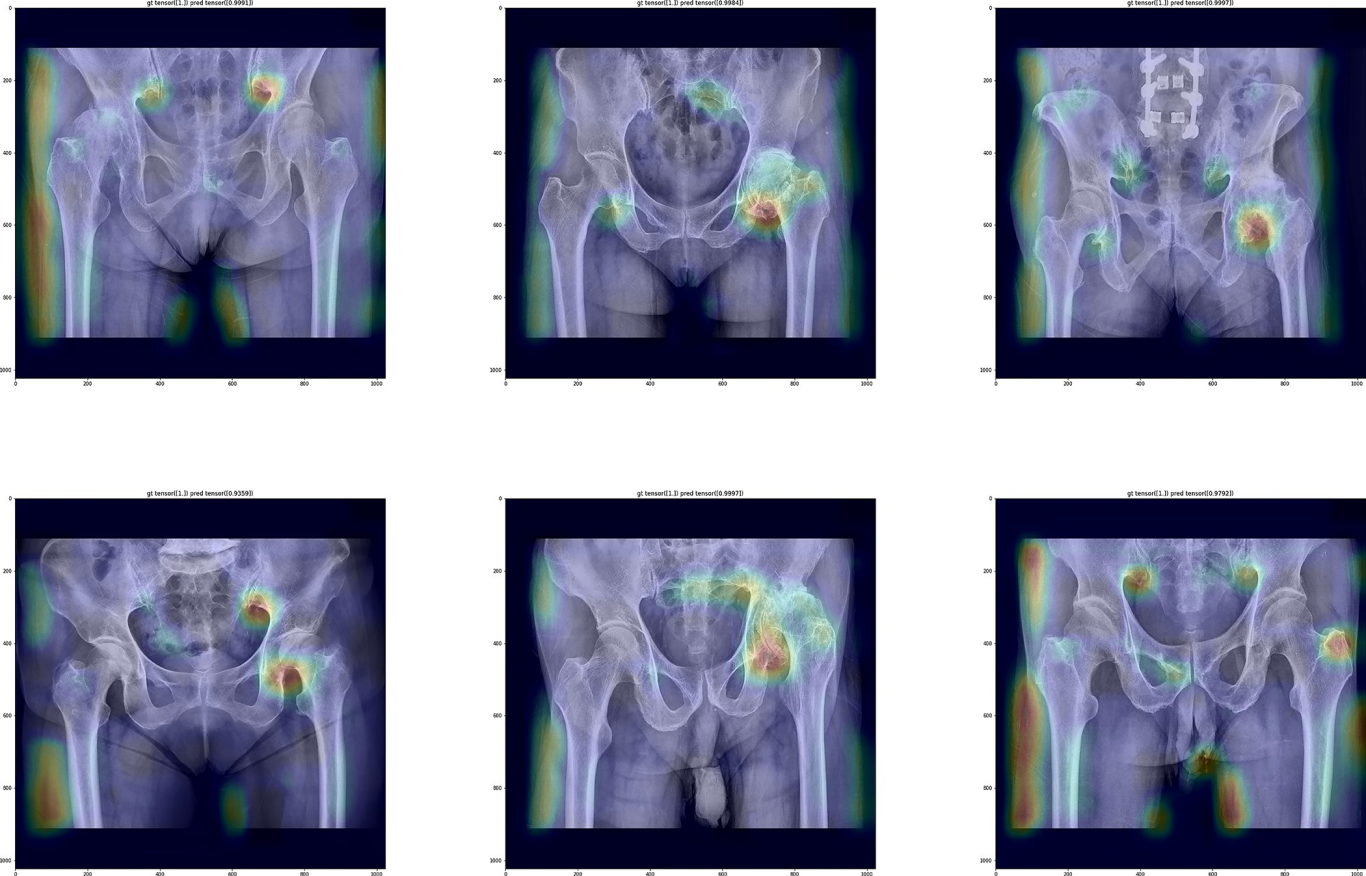
The Grad-CAM images of the 3^rd^ model showing the emphasis on sciatic notches.

The third model in which the preprocessed radiographs were used both for training set and the test set showed the accuracy, sensitivity, specificity, and F1-score of 0.92, 0.89, 0.98, and 0.94, respectively. The gradcam image showed that the third model mainly focused on the sciatic notch rather than the hip joint (Fig 3).

**Fig 4:**
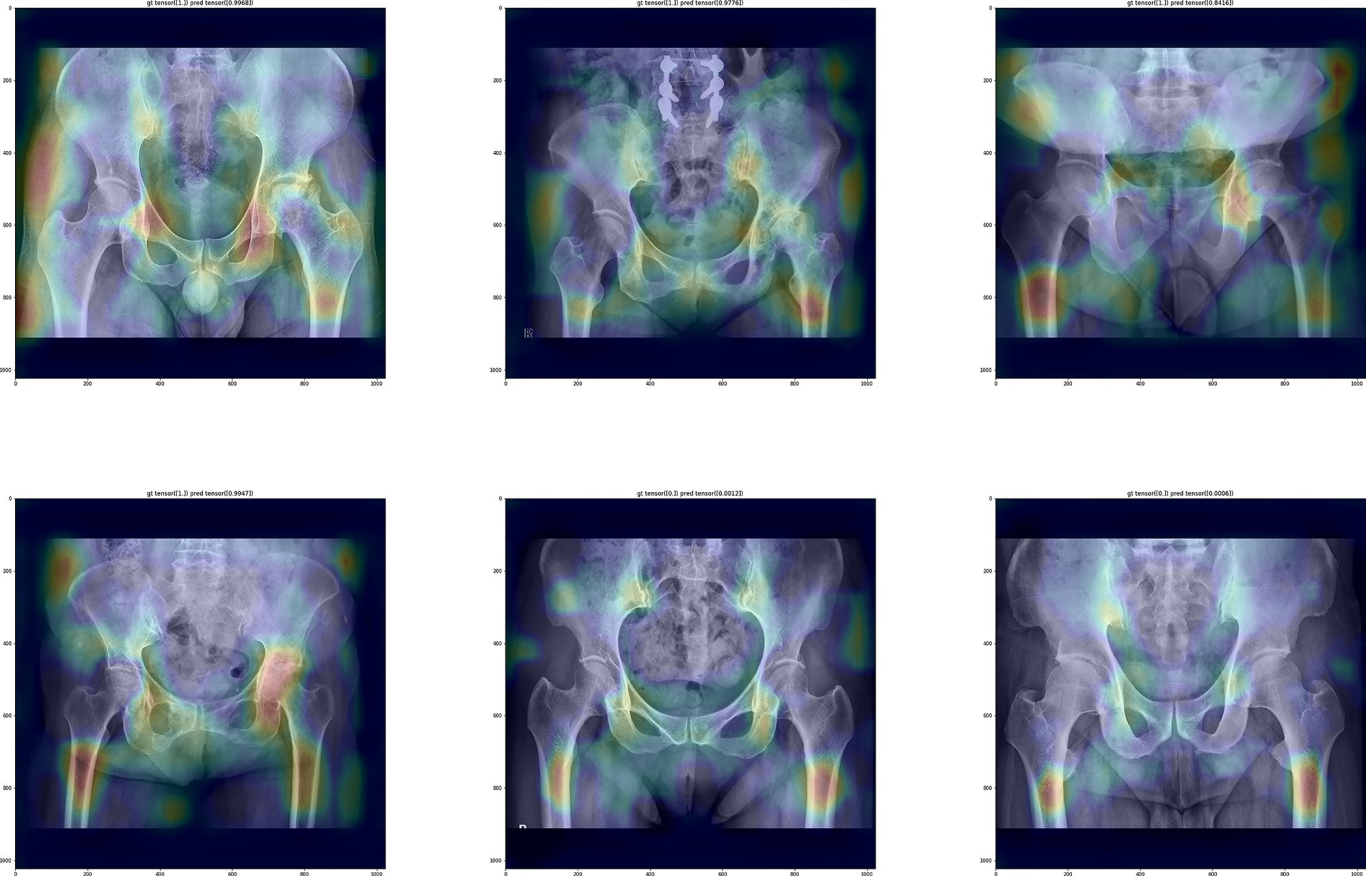
The Grad-CAM images of the 4^th^ model, which showed highest performance.

The fourth model in which the backbone algorithm was changed from DenseNet to EfficientNet with the addition of nonlocal block, showed the accuracy, sensitivity, specificity, and F1-score of 0.96, 0.96, 0.97, and 0.97, respectively (Fig 4).

**Fig 5:**
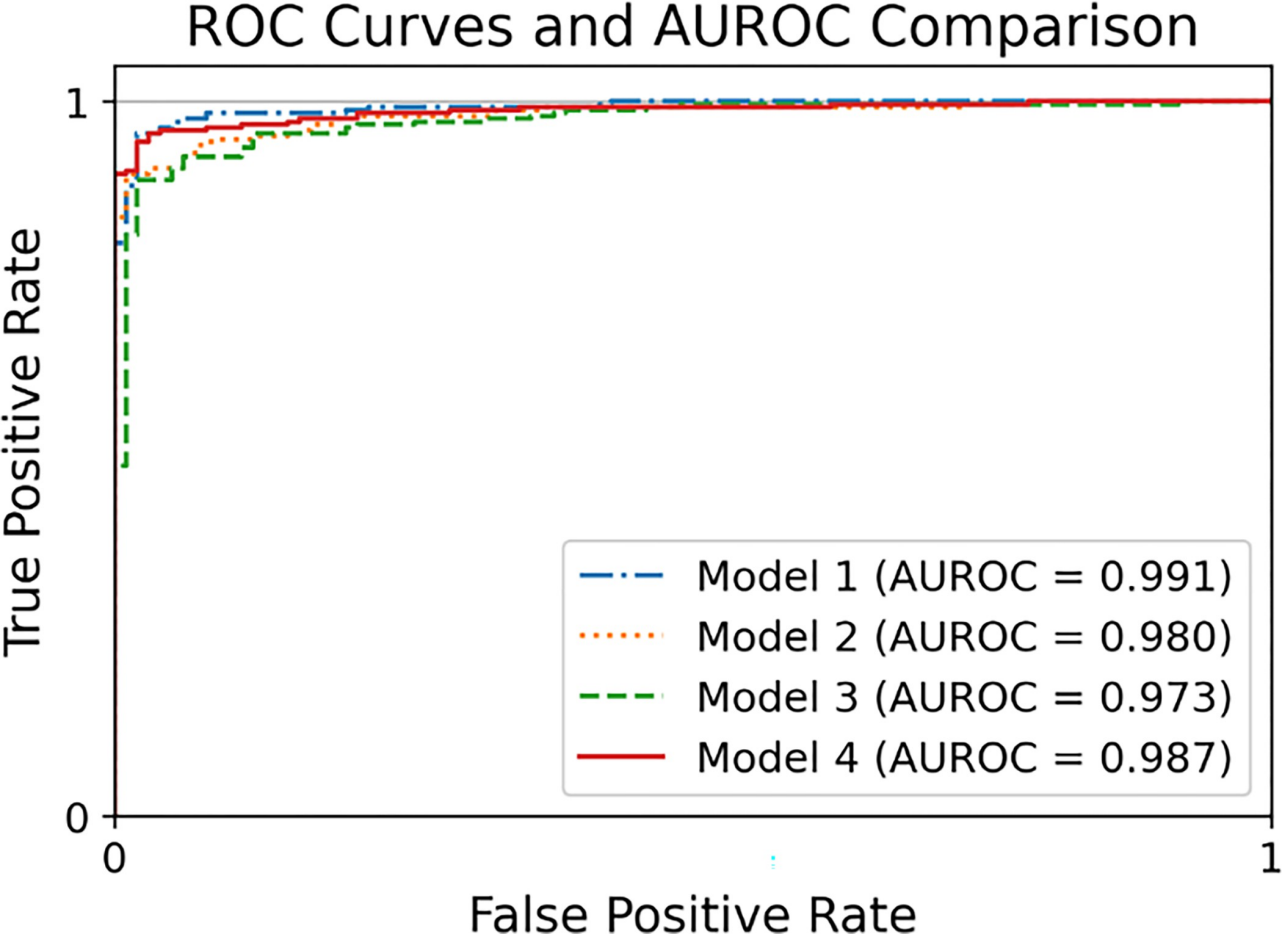
The Receiver Operating Characteristic (ROC) curve for each screening model.

The outcomes of the four models are summarized in Table 3 and Fig 5.

**Table 3:** Performance of four screening models for hip disease.

	1^st^ model	2^nd^ model	3^rd^ model	4^th^ model	External validation
**Accuracy**	0.96	0.93	0.92	0.96	0.94
**Sensitivity**	0.96	0.90	0.89	0.96	0.93
**Specificity**	0.98	0.99	0.98	0.97	0.96
**PPV**	0.99	0.99	0.99	0.99	0.95
**NPV**	0.90	0.80	0.78	0.90	0.93
**F1-score**	0.97	0.94	0.94	0.97	0.94
**AUROC**	0.99	0.98	0.97	0.99	0.98

PPV, positive predictive value; NPV, negative predictive value; AUROC, area under the receiver operating characteristic curve.

### External validation

There were 73 (37%) men in the normal group and 62 (31%) men in the hip disease group. The mean age of patients in normal group and hip disease group was 63.4 ± 9.8 years and 62.4 ± 16.1 years, respectively. The accuracy, sensitivity, specificity, F1 score, and AUROC of the final model was 0.94, 0.93, 0.96, 0.94, and 0.98, respectively.

## Discussion

The fourth screening model for hip disease developed in this study demonstrated very high performance with an AUROC of 0.99. External validation also confirmed the model’s outstanding performance with an AUROC of 0.98. This is a very encouraging outcome, especially considering that the diagnostic accuracy of plain hip radiographs has been historically limited [19].

Recently, deep learning-based diagnostic models for certain diseases have shown favorable results (Table 4) [30–40]. Most of the studies have focused on detection of the specific conditions such as osteoporosis [31,32,34,35,38,39,41] or fractures [30,36,40,42,43]. The prominent performance of deep learning-based models in diagnosis was abundantly studied using the chest radiograph, which is one of the most commonly taken radiographs [30,34,39,44]. Guo L. et al suggested that the diagnostic performance of chest radiographs were enhanced in recognition of various disease of the chest region with the AI assistance [44]. In 2022, Chae HD et al. found that the deep learning model and observers showed AUC of 0.85 and 0.84, respectively (p = 0.739) in the task of detecting cervical ossification of the posterior longitudinal ligament in plain radiographs [45]. In current study, we also found outstanding performance of screening model based on deep learning based algorithm. In fact, the performance of the developed screening model was even better than the previously reported diagnostic models because the aim of this model was to screen and differentiate only whether the patient has the hip disease or not rather than specifically classifying the diagnoses.

**Table 4:** The studies using plain radiographs and deep-learning algorithm for screening.

Author	Year	Condition	Algorithm	Radiograph	Performance
Ho et al.	2021	Osteoporosis	ResNet18	Pelvis	Sensitivity: 0.72 Specificity: 0.92
Hsieh et al.	2021	Osteoporosis	VGG16	Pelvis/Lumbar	AUC 0.97(pelvis) AUC 0.92(lumbar)
Jang R et al.	2021	Osteoporosis	VGG16	Pelvis	Sensitivity: 0.79 Specificity: 0.86 AUC 0.7
Jang M et al.	2022	Osteoporosis	OsPor-screen model	Chest	Sensitivity: 0.84 Specificity: 0.82 AUC 0.91
Lee et al.	2020	Osteoporosis	VGG16	Dental	Sensitivity: 0.90 Specificity: 0.81 AUC 0.86
Sato et al.	2022	Osteoporosis	ResNet50	Chest	Sensitivity: 0.81 Specificity: 0.74 AUC: 0.84
Mao L et al.	2022	Osteoporosis	DenseNet	Lumbar AP/Lat.	Sensitivity: 0.85 Specificity: 0.87 AUC: 0.94
Chae HD et al.	2022	Cervical OPLL	CNN	Lat. cervical	AUC 0.911
Ito S et al.	2023	Thoracic OPLL	YOLOv4	Lat. thoracic	Accuracy: 0.83 Precision 0.73 F-measure: 0.83
Hong N et al.	2023	Vertebral fracture	Deep CNN	Lat. spine	Accuracy: 0.91 Sensitivity: 0.76 Specificity: 0.94 AUC 0.93 F1-score: 0.91
Kim JH et al.	2021	Ankle fracture	Inception V3 CNN	Ankle AP/Lat.	AUC: 0.91 (AP) AUC: 0.95 (Lat.)
Cheng CT et al.	2024	Rib & clavicle fracture	DenseNet-121	Chest	Accuracy: 0.84 Sensitivity: 0.87 Specificity: 0.80 AUC: 0.91
Yoon AP et al.	2021	Scaphoid fracture	Deep CNN	Wrist	Sensitivity: 0.87 Specificity: 0.92 AUC 0.96
Gao Y et al.	2023	Hip fracture	DenseNet121	PXR	Accuracy: 0.96 Sensitivity: 0.94 Specificity: 0.96 AUC: 0.99
Magneli M et al.	2024	Hip dysplasia	VGG16	Pelvis	Precision: 90.3–96.8%
Current study		Hip disease	EfficientNet	Hip	Accuracy: 0.99 Sensitivity: 0.99 Specificity: 0.99 F1-score: 0.99 AUC:

OPLL, ossification of posterior longitudinal ligament; CNN, convolutional neural network; Lat., lateral; AUC, area under curve; ResNet, residual neural network; VGG, visual geometry group; DenseNet, densely connected convolutional networks; AP, anteroposterior; YOLOv4, you only look once version 4; PXR, frontal pelvic radiograph.

The screening models on a wide range of conditions by the plain radiographs are not commonly reported compared to the detection of the specific diagnosis. This may be due to the broader range of target diseases potentially leading to lower performance in a clinical setting, which could hinder its clinical utility [46]. However, binary classification on only the presence of diseases—not diagnosing each—could be a feasible task. In 2019, Hwang et al. used a deep learning algorithm to identify abnormal chest radiographs and reported AUC of 0.95, sensitivity of 0.89, and specificity of 0.70 [47]. The authors suggested that this screening model could inform physicians and radiologists of crucial diseases that might require urgent diagnosis and management [47]. More recently, Niehues et al. used bedside chest radiographs of the patients in intensive care unit to screen 8 findings (cardiac congestion, pleural effusion, air-space opacification, pneumothorax, central venous catheter, thoracic drain, gastric tube, and tracheal tube/cannula) and reported AUC of 0.85 to 0.99 [48]. Unlike the diagnosis of a specific disease, these studies are conducted regarding certain clinical situations such as emergency departments or intensive care units [47,48]. The screening model developed in the present study could be useful in the smaller medical institutions where the hip specialists are not present. After the screening, the patients who require further evaluation and timely appropriate management could be referred to hip specialists, reducing the possibility of misdiagnosis and the delay of diagnosis.

There are a few limitations to this study. First, the hospitals where the investigation was performed were tertiary referral hospitals and the diagnoses of the hip disease might differ from those of the patients in local hospitals. However, the radiographs of the various conditions used to develop the screening model could enhance the sensitivity, which is the main advantage for the screening model. Second, the scores of external validation were slightly lower than those of the internal validation. This might be due to the overfitting of the internal cohort [49]. However, the scores of the external validation were still excellent, indicating the high generalizability of the developed screening method.

In conclusion, the screening model developed in this study showed excellent performance both in internal and external validation. This screening model for hip disease is expected to be highly beneficial for primary physicians treating patients who present with hip pain in clinical settings. Building on the results of this study, future work could focus on developing more advanced diagnostic models capable of automatically classifying specific hip diseases using deep learning algorithms on plain radiographs. Long-term clinical validation across diverse populations and healthcare settings is also essential to ensure the generalizability and practical application of these models in real-world clinical environments.

## Supporting information

S1 Fig Architecture of the proposed deep learning model for hip disease screening. The model consists of an EfficientNet-B3 backbone for feature extraction, followed by a nonlocal block for capturing long-range dependencies. The extracted features are then processed through global average pooling, a fully connected layer, and a sigmoid activation for binary classification of normal vs. abnormal hip radiographs.
(TIF)

S1 File Model architectures. (DOCX)
