# Supplementary figures and images for: Deep learning based screening model for hip diseases on plain radiographs
Source: PLoS One. 2025 Feb 13;20(2):e0318022. doi: 10.1371/journal.pone.0318022 (PMC11825046; doi:10.1371/journal.pone.0318022)

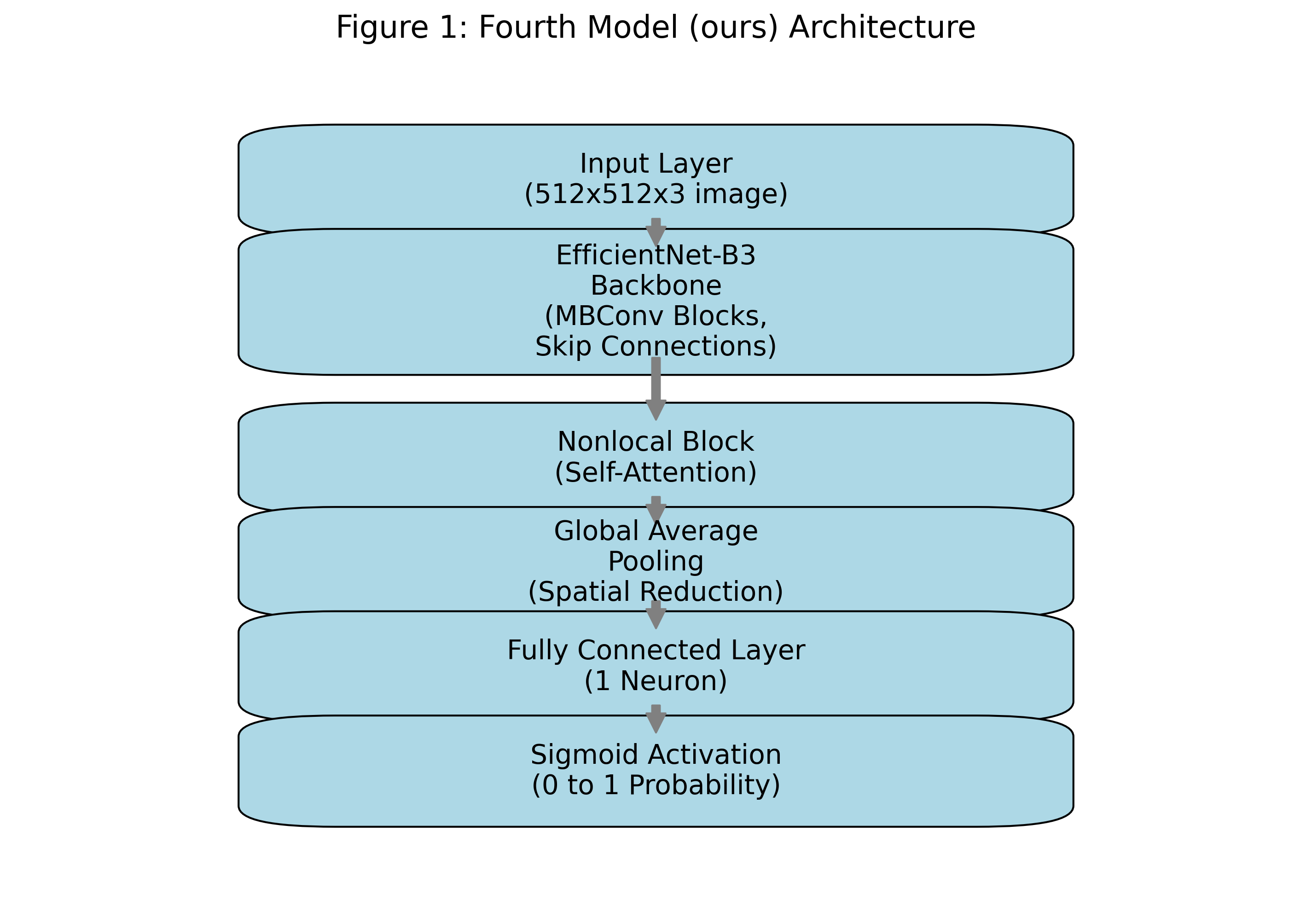

Supplement: S1 Fig — The model consists of an EfficientNet-B3 backbone for feature extraction, followed by a nonlocal block for capturing long-range dependencies. The extracted features are then processed through global average pooling, a fully connected layer, and a sigmoid activation for binary classification of normal vs. abnormal hip radiographs. (TIF) [file pone.0318022.s001.tif]
