# Supplementary material for: Deep learning based screening model for hip diseases on plain radiographs
Source: PLoS One. 2025 Feb 13;20(2):e0318022. doi: 10.1371/journal.pone.0318022 (PMC11825046; doi:10.1371/journal.pone.0318022)
Supplement: S1 File — (DOCX) [file pone.0318022.s002.docx]

**S1 File. Model architectures**

First model: DenseNet model using raw DICOM files as input:

This initial model used a DenseNet architecture and was trained and tested on raw DICOM image files without any preprocessing. The DenseNet architecture is known for its dense connectivity pattern between layers, which helps in feature reuse and efficient training.

Second model: Model trained on preprocessed images but tested on raw images: For this model, the training images were preprocessed using a series of augmentation techniques including center cropping, padding, resizing, and various image transformations (e.g., flipping, brightness adjustments, blurring). However, the test set used raw images without these preprocessing steps. This approach was likely used to test the model's generalization capabilities.

Third model: Model using preprocessed images for both training and testing: This model applied the same preprocessing techniques to both the training and test sets. The preprocessing included center cropping to 800x1024, padding to 1024x1024, resizing to 512x512, and various data augmentation techniques such as flipping, brightness adjustments, and geometric transformations.

Fourth model: Final model using EfficientNet backbone with a nonlocal block: The final and best-performing model used an EfficientNet-B3 architecture as the backbone. EfficientNet is known for its balanced trade-off between model size and accuracy. This model was enhanced with the addition of a nonlocal block after the main feature extraction layers. The nonlocal block is designed to capture long-range dependencies in the image, which can be particularly useful for analyzing complex medical images. This model also used the same preprocessing and augmentation techniques as the third model.

All models were trained using binary cross-entropy loss and the Adam optimizer. The training process included techniques to handle class imbalance, such as the ImbalancedDatasetSampler, which helped to ensure that both normal and abnormal cases were equally represented during training despite the imbalance in the original dataset.
